# Global landscape and knowledge structure of research on pancreatitis and gut microbiota: a bibliometric analysis based on WoSCC and Scopus (2006 - 2025)

**DOI:** 10.3389/fcimb.2026.1878923

**Published:** 2026-07-24

**Authors:** Huichuan Tian, Liangyue Wang, Zixuan He, Guanlu Li, Ziyi He, Gang Wei

**Affiliations:** 1 Department of Emergency, Xi’an Hospital of Traditional Chinese Medicine, Xi’an, China; 2 Faculty of Chinese Medicine, Macau University of Science and Technology, Taipa, Macao SAR, China; 3 School of Clinical Medicine, Hubei University of Medicine, Shiyan, Hubei, China

**Keywords:** bibliometric analysis, chronic pancreatitis, CiteSpace, dysbiosis, gut microbiota, microbial metabolites, pancreatitis, VOSviewer

## Abstract

**Background:**

The research on pancreatitis and gut microbiota has expanded rapidly in recent years. Growing evidence suggests that gut dysbiosis may play an important role in pancreatic inflammation, intestinal barrier dysfunction and disease progression, but global research landscape, major contributors, knowledge structure and hotspots have not been fully defined.

**Materials and methods:**

Publications related to pancreatitis and gut microbiota were retrieved from the Web of Science Core Collection and Scopus. The search was conducted on Jan 5, 2026, and covered studies published between 2006 and 2025. Bibliometrix, VOSviewer, CiteSpace and Microsoft Excel were used to analyze the annual publication trends, journal sources, authors, countries, institutions, cited articles, co-cited references, keyword co-occurrence, thematic clustering, and time evolution.

**Results:**

The annual number of publications increased markedly from 2006 to 2025, with sustained acceleration after 2020. Pre- and post-2020 comparison suggested a shift from early clinical and intervention-oriented topics toward more mechanism-based and translational research, including gut microbiota, immune regulation, and multi-omics approaches. China contributed the most publications, followed by the United States. Citation and co-citation analyses identified major thematic groups related to acute pancreatitis, chronic pancreatitis, pancreatic homeostasis, dietary intervention, and microbiota-targeted research. Frequent keywords included human, gut microbiota, acute pancreatitis, intestinal flora, inflammation, dysbiosis, probiotics and chronic pancreatitis. Recent burst keywords were more closely related to bifidobacterium, short-chain fatty acids, metabolomics, acute lung injury, fecal analysis, and lipopolysaccharide, suggesting changing research attention toward mechanism-oriented and translational topics.

**Conclusion:**

This bibliometric analysis provides a detailed overview of global studies on pancreatitis and gut microbiota from 2006 to 2025. The field has expanded rapidly, and bibliometric patterns suggest increasing attention to mechanistic and translational investigations. Some highly co-cited references were related to pancreatic cancer, inflammatory bowel disease, microbial metabolites, immune regulation, and host-microbe interactions rather than pancreatitis itself, reflecting the broader knowledge base of this research field. Acute pancreatitis remained the most studied topic, while chronic pancreatitis, microbial metabolites, host response, and microbiota-targeted approaches gained increasing attention in recent years. Future research may shift from descriptive microbiota profiling to mechanism-oriented and translational studies.

## 1 Introduction

Pancreatitis comprises a group of inflammatory pancreatic disorders (Li et al., 2025; Xu et al., 2025). Acute pancreatitis is one of the most common gastrointestinal causes of hospital admission and ranges from mild self-limited inflammation to severe disease with persistent organ failure. Chronic pancreatitis is a progressive fibroinflammatory disease (Banks et al., 2013; Lee and Papachristou, 2019; Petrov and Yadav, 2019). It is often associated with chronic abdominal pain, exocrine pancreatic insufficiency, diabetes, malnutrition, and reduced quality of life (Olesen et al., 2017; Min et al., 2018; Bruni et al., 2025). While acute and chronic pancreatitis differ in clinical presentation/onset and disease course, both conditions involve complex interactions between inflammation/immune response, metabolic disturbance and extra-pancreatic complications. For many years, pancreatitis was mainly studied from the perspective of pancreatic injury, enzyme activation, acinar cell damage, and systemic inflammation (Chen F. et al., 2024; Gukovskaya et al., 2019; Lee and Papachristou, 2019; Saluja et al., 2019).

In recent years, the gut has become increasingly relevant to the study of pancreatitis. Gut microbiota is involved in mucosal barrier maintenance, immune regulation, microbial metabolite production, and host metabolic homeostasis (Gasaly et al., 2021; Liu et al., n.d.; Fu et al., 2022). These functions are directly relevant to several clinically important features of pancreatitis, including intestinal barrier dysfunction, bacterial translocation, infected necrosis, systemic inflammation, nutritional intolerance, and longer-term metabolic complications. For this reason, the relationship between pancreatitis and gut microbiota is not simply an extension of general microbiome research, but a disease-specific research area shaped by the pathophysiological links between intestinal ecology and pancreatic injury.

The connection between gut microbiota and acute pancreatitis has been studied more extensively than that with chronic pancreatitis. Severe acute pancreatitis is often accompanied by intestinal barrier dysfunction and systemic inflammatory response. Enteric bacteria have long been considered a potential source of infectious complications (Landahl et al., 2015; Agarwal et al., 2023). Early studies therefore focused largely on probiotics, synbiotics, and bacterial translocation. However, the PROPATRIA trial showed that probiotic prophylaxis did not reduce infectious complications and was associated with increased mortality in patients with predicted severe acute pancreatitis (Besselink et al., 2008; Bongaerts and Severijnen, 2016). This finding changed the way microbiota-based interventions were viewed in this field. Later studies began to focus more on microbial composition, dysbiosis, disease severity, host immune response, and metabolic changes. Recent studies have reported that gut microbiota dysbiosis may worsen the severity of acute pancreatitis in both patients and experimental models (Zhu et al., 2018; Ammer-Herrmenau et al., 2024). Compared with acute pancreatitis, the connection between chronic pancreatitis and gut microbiota has been less extensively studied, but it is becoming increasingly relevant. Chronic pancreatitis can alter digestion, nutrient absorption, bile acid metabolism, glucose homeostasis, and intestinal conditions through exocrine and endocrine pancreatic dysfunction (Bruni et al., 2025). These changes may reshape the intestinal microbial environment. Clinical studies have reported altered intestinal microbiota in patients with chronic pancreatitis, especially in those with diabetes and metabolic abnormalities (Zhu et al., 2018).

As research has increased, the literature on pancreatitis and gut microbiota has become increasingly diverse. A previous bibliometric analysis summarized pancreatic diseases and gut microbiota research from 2002 to 2022 (Li et al., 2024). However, that analysis did not specifically clarify how the knowledge structure of pancreatitis-related gut microbiota research has evolved in the most recent period, nor did it fully capture the rapid expansion of this field after 2020.

In the present study, we extended the observation window to 2025 and integrated records from both the Web of Science Core Collection and Scopus to provide a broader and more up-to-date overview of the field. More importantly, because the annual publication curve showed a sustained acceleration from 2020 onward, we further compared the pre-2020 and post-2020 periods to examine whether the thematic structure and emerging hotspots of this field have changed over time. This design enabled us not only to update publication trends, but also to identify temporal shifts in the knowledge base, research focus, and developmental trajectory of pancreatitis and gut microbiota research. Bibliometric analysis is a useful way to map the development of a research field (Chen, 2006; Aria and Cuccurullo, 2017).

## 2 Materials and methods

### 2.1 Data sources and search strategy

The bibliographic data for this study were retrieved from the Web of Science Core Collection (WoSCC) and Scopus. These two databases were selected because they provide broad coverage of biomedical and interdisciplinary literature and are commonly used in bibliometric research. The search strategy was designed to identify publications related to pancreatitis and gut microbiota.

To improve retrieval sensitivity, the original search included multiple terms related to pancreatitis and intestinal microbiota. Because the terminology used in this field is heterogeneous, broad expressions were retained at the retrieval stage to reduce the risk of missing potentially relevant studies. However, we recognized during revision that some terms in the original strategy were broad or suboptimal in specificity. Therefore, all retrieved records underwent additional manual eligibility screening to ensure that the final dataset remained focused on pancreatitis and gut microbiota research.

The search was performed on January 5, 2026, and covered publications from 2006 to 2025. Records indexed as 2026 publications were excluded to maintain a consistent observation window. Only original articles and review articles were included. The document-type restriction was applied during database retrieval by selecting Article and Review Article in WoSCC and Article and Review in Scopus before record export. Meeting abstracts, editorials, letters, corrections, book chapters, conference papers, and records with incomplete bibliographic information were excluded. The complete original search strategies used for WoSCC and Scopus are provided in **Supplementary Table 1**.

### 2.2 Data preprocessing and bibliometric analysis

After retrieval, full bibliographic records and cited references were exported from the WoSCC and Scopus in plain text and CSV formats. The records from both databases were imported into the R package Bibliometrix for preprocessing and bibliometric analysis. Duplicate records were first identified using DOI, title, author names, journal name, and publication year in Bibliometrix. Records without DOI were further checked manually according to title, first author, source title, publication year, and, when necessary, abstract information.

**Figure 1:**
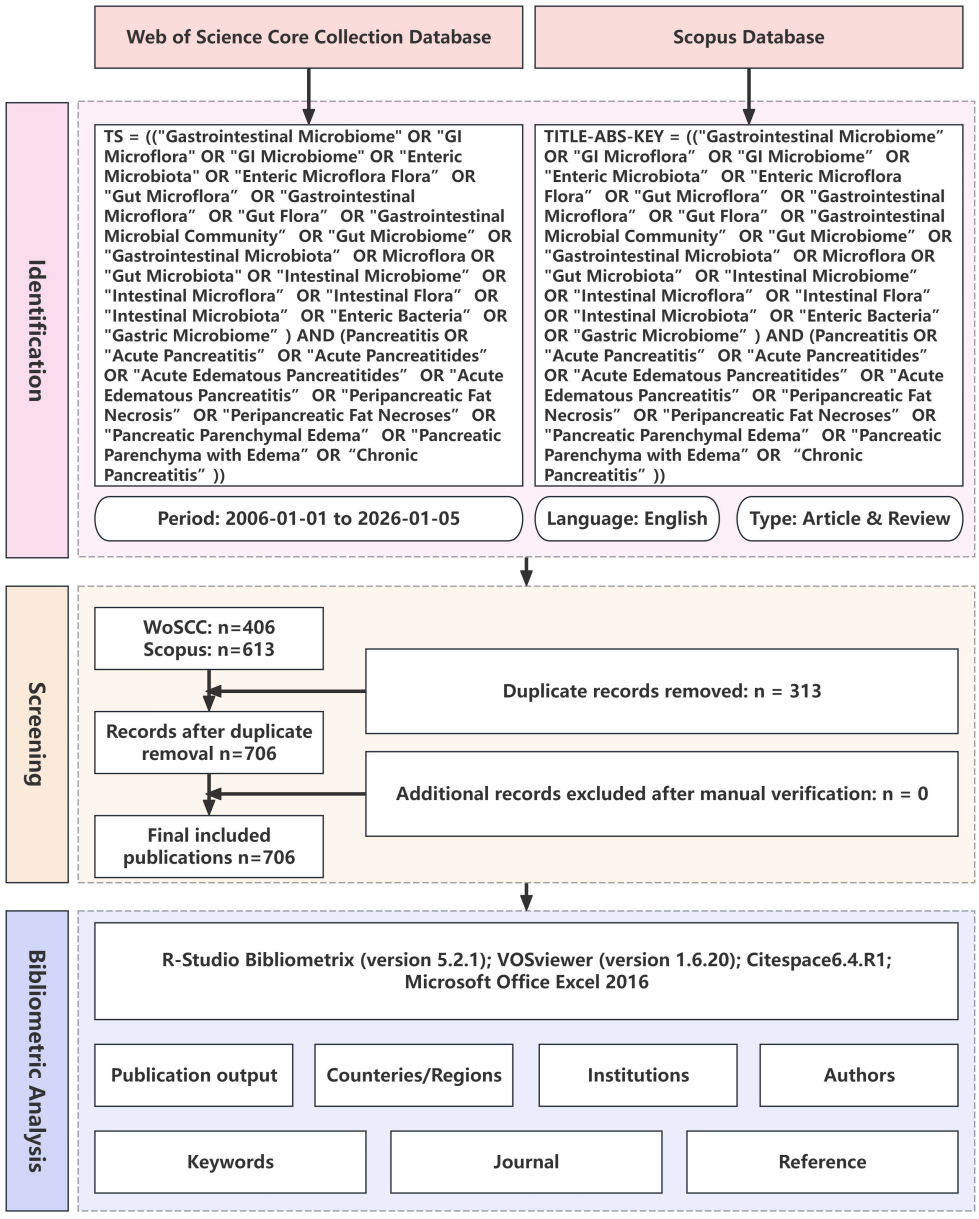
Study selection and bibliometric analysis workflow.

After deduplication, the remaining 706 records were manually checked for eligibility based on titles and abstracts, and full texts were checked when relevance remained uncertain. This screening step was used to verify that the deduplicated records were relevant to pancreatitis and gut microbiota research, rather than to apply an additional quantitative exclusion stage. Publications were considered eligible when pancreatitis and gut microbiota-related topics constituted a main focus of the study. Eligible topics included pancreatitis, acute pancreatitis, chronic pancreatitis, pancreatic inflammatory conditions, gut microbiota, gut microbiome, intestinal flora, microbial dysbiosis, probiotics, microbial metabolites, and microbiota-targeted intervention. No additional records were excluded after manual eligibility verification; therefore, the final bibliometric dataset contained 706 records (**Figure 1**). Two reviewers (LY W and GL L) independently verified the eligibility of the deduplicated records according to the predefined criteria. Titles and abstracts were first reviewed, and full texts were checked when relevance remained uncertain. Disagreements were resolved through discussion, and a third reviewer (HC T) was consulted when consensus could not be reached. No formal inter-reviewer agreement statistic was calculated because the screening was used to construct a bibliometric dataset rather than to perform effect-size synthesis; however, all uncertain records were reviewed jointly before final inclusion.

The cleaned dataset was used to calculate the main bibliometric indicators, including annual publication output, average citations per document, journal productivity, author productivity, institutional contribution, country-level output, total citations, co-citation frequency, H-index, G-index, and collaboration link strength. Source analysis was performed to identify productive and influential journals. Author, country, and institutional collaboration analyses were conducted to describe the collaborative structure of the field. Citation and co-citation analyses were used to identify highly cited articles, influential co-cited references, and the intellectual base of pancreatitis and gut microbiota research. It should be noted that co-cited references were extracted from the reference lists of the included source records. Therefore, the eligibility screening criteria were applied to the source records included in the bibliometric dataset, but not to every cited reference appearing in the co-citation analysis.

Bibliometric visualization was conducted using VOSviewer, CiteSpace, and Bibliometrix or Biblioshiny. VOSviewer was used to construct co-authorship networks, co-citation networks, keyword co-occurrence maps, and density maps. In VOSviewer density visualizations, colors reflect the local density of items based on node weights and the distribution of neighboring nodes. Therefore, density maps were used to identify areas with high local concentration of related items, rather than to provide an independent ranking of publication output, citation impact, or collaboration strength. CiteSpace was used for reference clustering, citation burst detection, keyword burst detection, and temporal evolution analysis. In CiteSpace, the time span was set from 2006 to 2025, with one year per slice. The selection criterion was set as g-index with k = 25. The link retaining factor was 2.5, the maximum look back years was 5, and the e value was set to 1.0. No pruning strategy was applied. The generated network contained 644 nodes and 426 links, with a network density of 0.0206. The largest connected component contained 572 nodes, accounting for 88% of the network. Cluster quality was evaluated using modularity Q and weighted mean silhouette values. The modularity Q was 0.4688, and the weighted mean silhouette value was 0.7833, indicating that the clustering structure was acceptable and that the clusters had good internal consistency. Country-level collaboration patterns were visualized using VOSviewer, whereas the corresponding author’s country distribution and SCP/MCP classification were obtained using Bibliometrix.

Microsoft Excel was used for data organization, descriptive statistics, and plotting of annual publication trends and source-level indicators. For country- and institution-level analyses, affiliation information was analyzed according to the indexed names provided by the source databases. University systems, hospitals, research institutes, and single-campus institutions were retained as indexed affiliation entities when they appeared in the original records. The institutional analysis was used to describe collaboration patterns among indexed affiliation entities rather than to generate a fully normalized ranking of independent institutions.

### 2.3 Keyword co-occurrence, clustering, and temporal evolution analysis

Keyword-based analyses were performed to describe the thematic structure and temporal evolution of research on pancreatitis and gut microbiota. The keyword sources included author-supplied keywords and database-indexed keywords. Before analysis, synonymous terms and spelling variants were checked and harmonized where appropriate. Terms such as “gut microbiota,” “gut microbiome,” “intestinal microbiota,” and “intestinal flora” were reviewed to reduce unnecessary fragmentation while preserving the original meaning of the indexed records. The generic terms such as “human,” “nonhuman,” “animals,” and “mouse” were not part of the search strategy but appeared in the keyword analysis because database-indexed keywords were included together with author-supplied keywords. These terms mainly reflect indexing categories or study types rather than substantive research themes. Therefore, high-frequency generic terms were interpreted cautiously and were not treated as equivalent to disease-, mechanism-, or microbiota-related thematic keywords.

Keyword frequency analysis was first conducted to identify the most commonly used terms in the field. A keyword word cloud was generated to provide an overview of high-frequency terms. Annual keyword distribution was then analyzed to show changes in the use of major keywords from 2006 to 2025. Keyword co-occurrence analysis was performed to examine the relationships among high-frequency terms. In VOSviewer, keywords were treated as nodes, and co-occurrence links represented the frequency with which two keywords appeared in the same publication. Node size reflected keyword frequency, while link thickness represented co-occurrence strength. Keyword clustering was used to identify major thematic groups in the field. Density visualization was further applied to show areas with frequent and closely connected keywords. The year 2020 was selected as the dividing point because the annual publication curve showed sustained acceleration from 2020 onward. This cutoff was therefore used as a data-driven temporal boundary for exploratory comparison rather than as a predefined biological or clinical turning point. We acknowledge that alternative cutoff years might produce somewhat different thematic patterns.

## 3 Results

### 3.1 Annual publications and average citations

**Figure 2:**
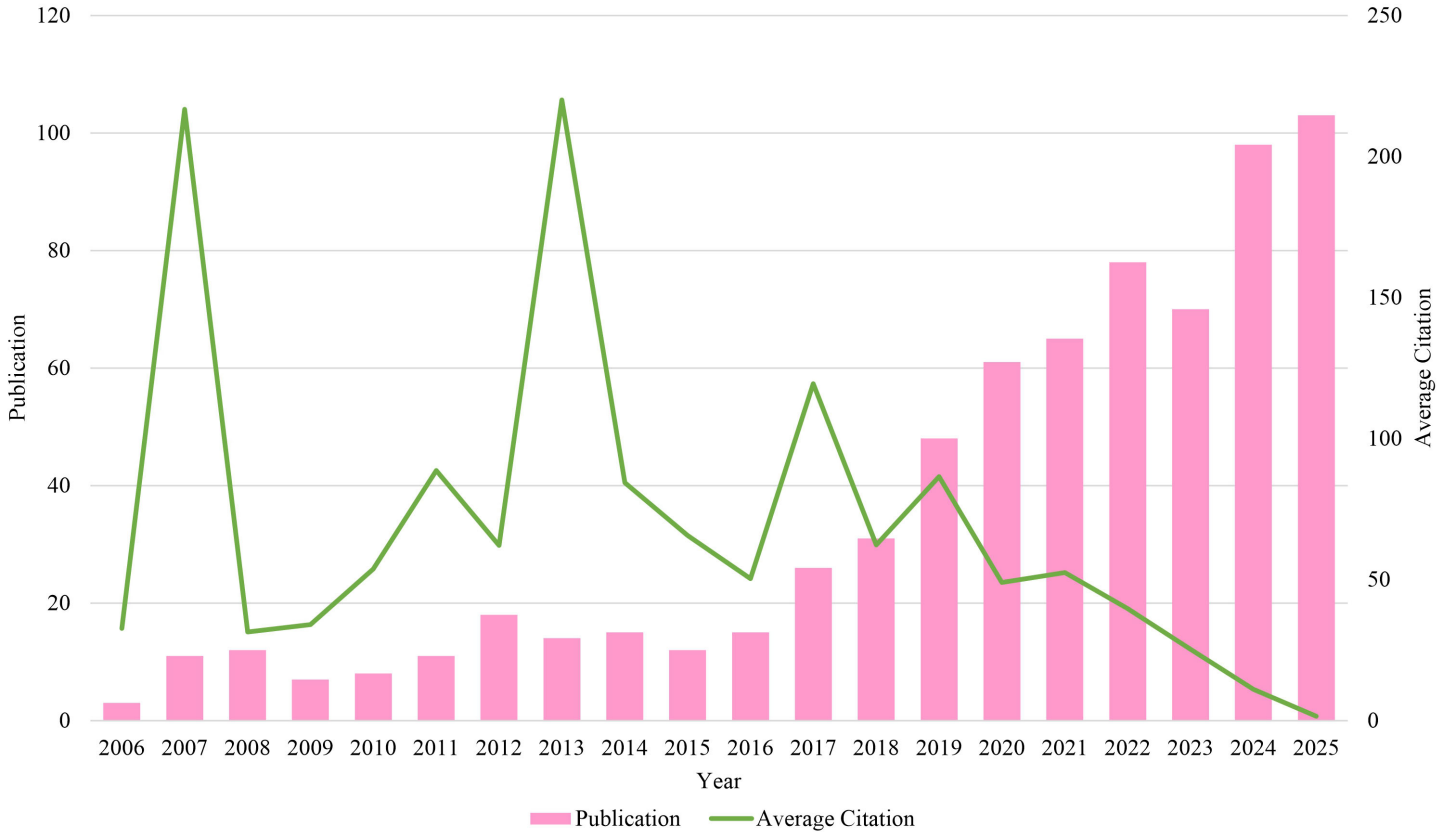
Trends in annual publications and average citations of studies related to pancreatitis and gut microbiota between 2006 and 2025. The pink bars represent the annual number of publications, and the green line indicates the average citations per publication.

Between 2006 and 2025, the annual number of publications on pancreatitis and gut microbiota increased markedly (**Figure 2**). From 2006 to 2017, the annual output remained at a relatively low but fluctuating level, followed by a rapid exponential growth after 2018. This increase became more sustained from 2020 onward, suggesting that the field entered a phase of accelerated development in recent years. The highest annual publication output was reached in 2025. In contrast, the average citation per article varied substantially across years and was generally higher in the earlier period, particularly in 2007 and 2013, indicating the strong influence of several early publications. The lower average citation values in the most recent years should be interpreted with caution, because newly published articles have had less time to accumulate citations.

### 3.2 Sources

**Figure 3:**
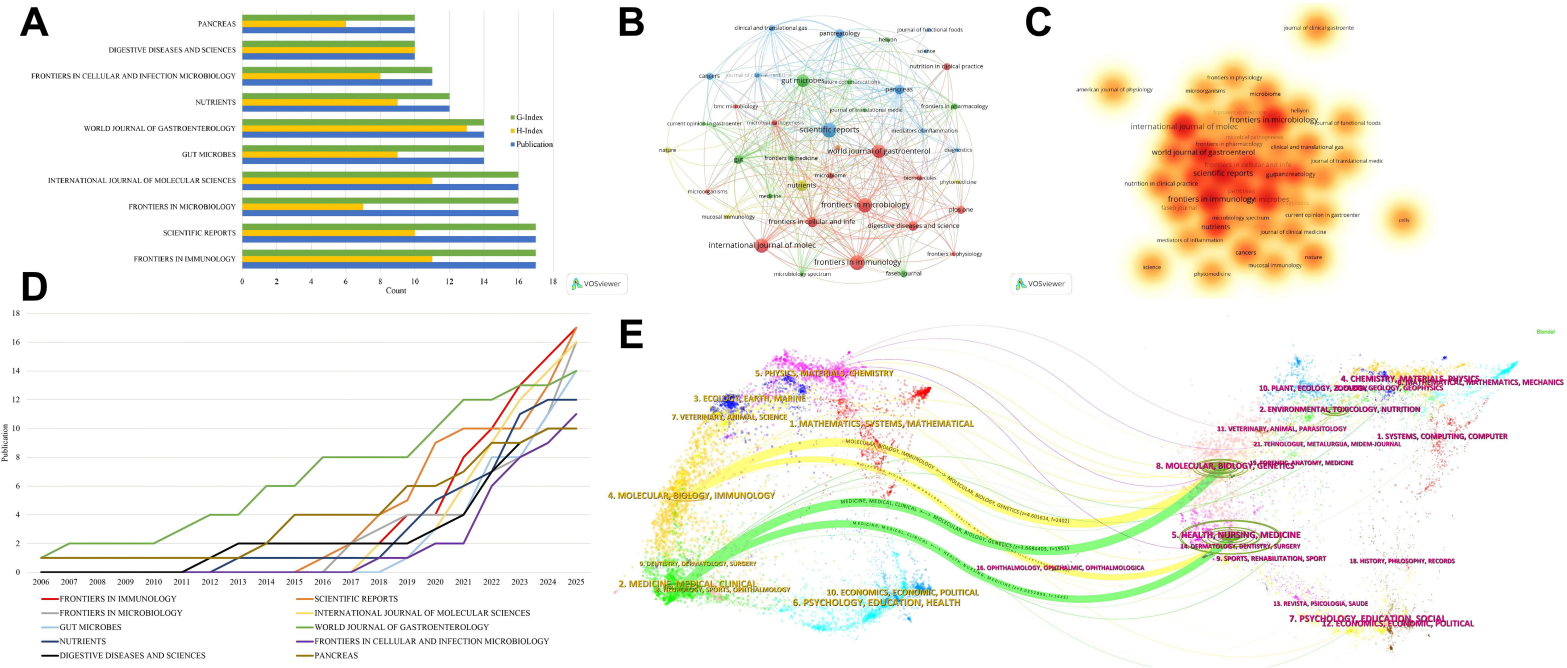
Source analysis of publications on pancreatitis and gut microbiota. **(A)** Publication volume, H-index, and G-index of the top 10 journals. **(B)** co-citation network map of influential journals. **(C)** Density map of influential journals. **(D)** Annual publication trends of the top 10 journals. **(E)** Dual-map overlay analysis of citing and cited journals.

**Figure 3A** and **Table 1** summarize the top 10 journals publishing research on pancreatitis and gut microbiota. *Frontiers in Immunology*, *Scientific Reports*, *Frontiers in Microbiology*, and *International Journal of Molecular Sciences* were among the most productive sources. *Gastroenterology*-related journals, including *Gut Microbes*, *World Journal of Gastroenterology*, *Digestive Diseases* and *Sciences*, and *Pancreas*, also made important contributions. In addition, journals with relatively high H-index and G-index values, such as *Frontiers in Immunology*, *Scientific Reports*, *Frontiers in Microbiology*, *International Journal of Molecular Sciences*, and *World Journal of Gastroenterology*, demonstrated strong academic influence in this field.

**Table 1:** Top 10 sources in the research related to pancreatitis and gut microbiota.

Rank	Source	Publication	H-index	G-index	Citation
1	*FRONTIERS IN IMMUNOLOGY*	17	11	17	651
2	*SCIENTIFIC REPORTS*	17	10	17	542
3	*FRONTIERS IN MICROBIOLOGY*	16	7	16	321
4	*INTERNATIONAL JOURNAL OF MOLECULAR SCIENCES*	16	11	16	643
5	*GUT MICROBES*	14	9	14	651
6	*WORLD JOURNAL OF GASTROENTEROLOGY*	14	13	14	1508
7	*NUTRIENTS*	12	9	12	574
8	*FRONTIERS IN CELLULAR AND INFECTION MICROBIOLOGY*	11	8	11	242
9	*DIGESTIVE DISEASES AND SCIENCES*	10	10	10	250
10	*PANCREAS*	10	6	10	249

The journal co-citation network and density map showed that *World Journal of Gastroenterology*, *Scientific Reports*, *Gut Microbes*, *International Journal of Molecular Sciences*, *Frontiers in Microbiology*, and *Frontiers in Immunology* occupied prominent positions in the journal co-citation structure (**Figures 3B, C**). The annual outputs of the top 10 journals increased markedly after 2019, suggesting that attention to the association between pancreatitis and gut microbiota was growing rapidly across journals (**Figure 3D**).

The dual-map overlay analysis showed that citing journals were mainly distributed in medicine, medical, clinical research, molecular biology, and immunology, whereas cited journals were mainly concentrated in molecular biology, genetics, health, medicine, environmental toxicology, and nutrition (**Figure 3E**). These results indicate that pancreatitis and gut microbiota research is supported by knowledge bases involving clinical medicine, gastroenterology, microbiology, immunology, molecular biology and nutrition.

### 3.3 Author collaboration analysis

**Figure 4:**
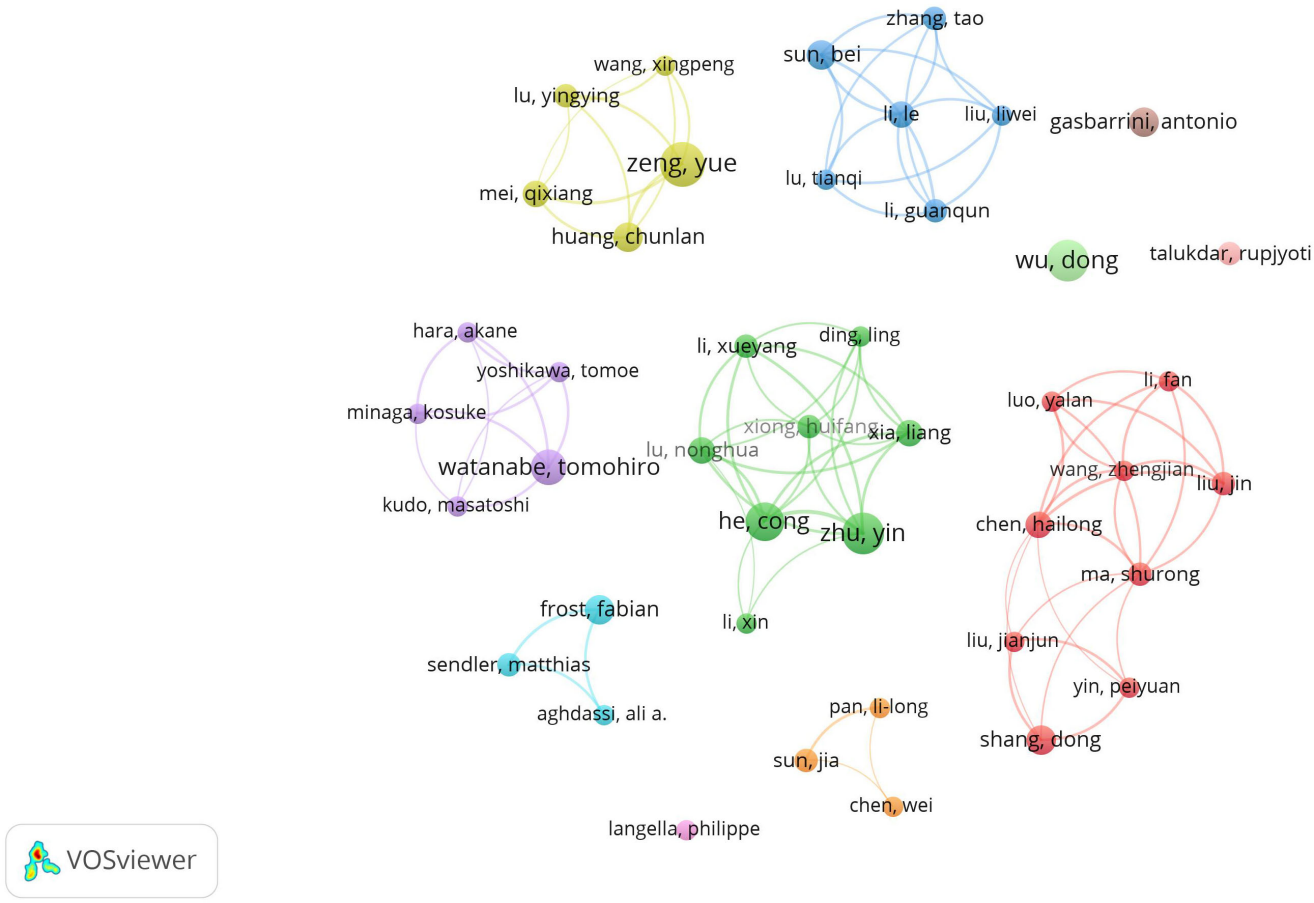
Co-authorship network map of influential authors in pancreatitis and gut microbiota research. Each node represents an author, and the node size indicates the author’s relative contribution or influence in the network. Links between nodes indicate co-authorship relationships, and different colors represent distinct collaboration clusters.

As shown in **Figure 4** and **Table 2**, the co-authorship network of influential authors displayed a distinct clustered pattern. Several major collaborative groups were identified, indicating that studies on pancreatitis and gut microbiota have been mainly conducted by relatively independent research teams. The largest clusters included author groups represented by He Cong, Zhu Yin, Xiong Huifan, Xia Liang, and Lu Nonghua; Chen Hailong, Wang Zhengjian, Liu Jin, Ma Shurong, and Shang Dong; Zeng Yue, Huang Chunlan, Mei Qixiang, and Wang Xingpeng; and Sun Bei, Zhang Tao, Li Le, Liu Liwei, and Li Guanqun.

**Table 2:** Top 10 productive authors in pancreatitis and gut microbiota research.

Rank	Author	Publication	Citations	Mean citations	Country
1	Zeng, Yue	13	492	37.85	China
2	Wu, Dong	12	270	22.50	China
3	Zhu, Yin	12	777	64.75	China
4	He, Cong	11	751	68.27	China
5	Watanabe, Tomohiro	10	466	46.60	Japan
6	Frost, Fabian	8	138	17.25	Germany
7	Gasbarrini, Antonio	8	220	27.50	Italy
8	Huang, Chunlan	8	220	27.50	China
9	Shang, Dong	8	107	13.38	China
10	Sun, Bei	8	228	28.50	China

The dense links within each cluster suggest close collaboration among authors from the same research group. However, relatively few links were observed between different clusters, indicating that inter-group collaboration remains limited. Several smaller or isolated author groups were also present, including those involving Watanabe Tomohiro, Frost Fabian, Gasbarrini Antonio, Talukdar Rupjyoti, and Langella Philippe. Overall, these results suggest that the field has formed a number of stable teams, but more general collaboration networks among institutions and countries are not fully established yet.

### 3.4 Regional distribution

**Figure 5:**
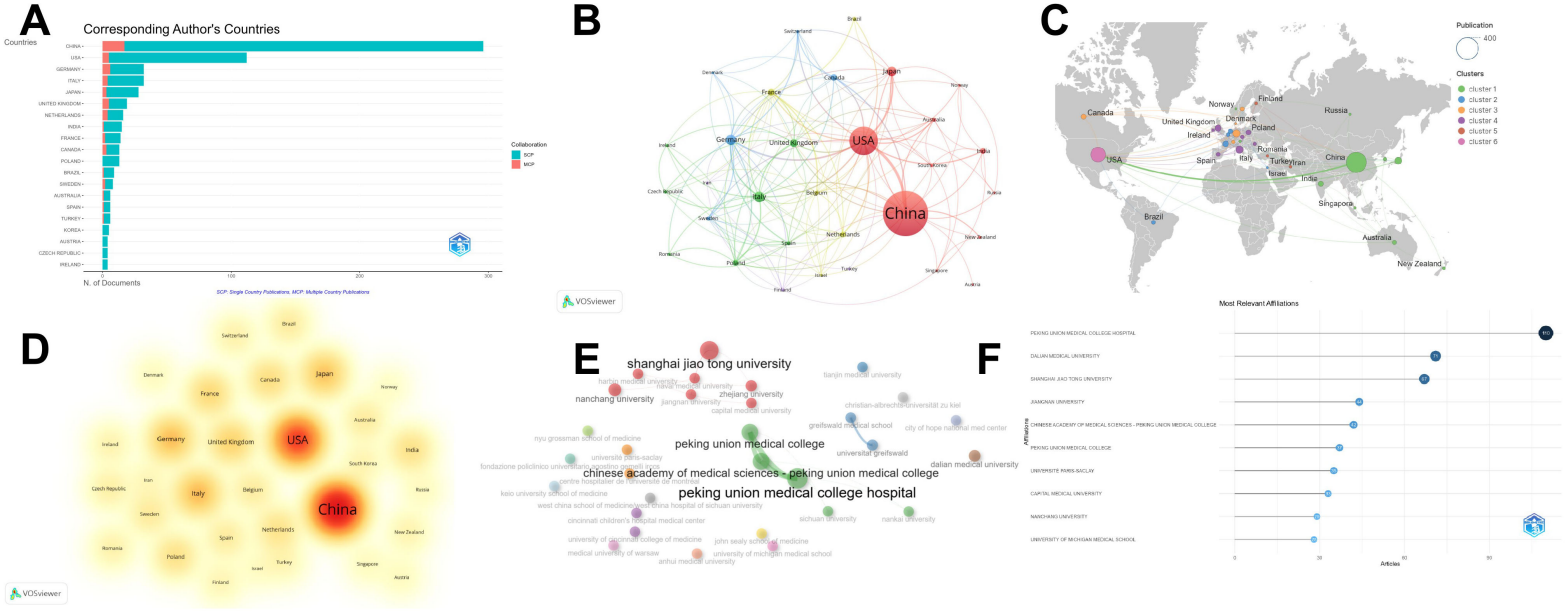
Regional distribution analysis of publications on pancreatitis and gut microbiota. **(A)** Corresponding author’s countries, showing the distribution of single-country publications (SCP) and multiple-country publications (MCP). **(B)** Country collaboration network map. **(C)** World map of publication output by influential countries. **(D)** Country collaboration density map. **(E)** Co-authorship network of indexed affiliation entities in this research field. **(F)** Most relevant affiliations.

Geographical analysis showed that publications on pancreatitis and gut microbiota were concentrated in a limited number of countries. China contributed the largest number of publications, followed by the United States, while Germany, Italy, Japan, the United Kingdom, the Netherlands, India, France, and Canada also made notable contributions (**Figure 5A**). In most countries, single-country publications accounted for a larger proportion than multiple-country publications, indicating that domestic collaboration remained more common than international collaboration.

The country collaboration network further distinguished collaboration structure from publication output. China and the United States occupied central positions with extensive international links, while several European countries, including Germany, Italy, the United Kingdom, France, and the Netherlands, also showed visible collaboration connections (**Figure 5B**). The world map indicated that the main research output was distributed across Asia, North America, and Europe (**Figure 5C**). The density map reflected areas with a high local concentration of country nodes and neighboring collaboration links based on VOSviewer density visualization, rather than a separate ranking of country productivity (**Figure 5D**).

At the institutional level, some leading affiliations were located in China (**Figure 5E**). Peking Union Medical College Hospital ranked first among the most relevant affiliations, followed by Dalian Medical University, Shanghai Jiao Tong University, Nanchang University, Chinese Academy of Medical Sciences and Peking Union Medical College, Peking Union Medical College, University Paris-Saclay, Capital Medical University, and University of Michigan Medical School (**Figure 5F**; **Table 3**). In the affiliation co-authorship network, Peking Union Medical College Hospital, Chinese Academy of Medical Sciences and Peking Union Medical College, Peking Union Medical College, Shanghai Jiao Tong University, and Nanchang University occupied relatively prominent positions. Because institutional names were retained according to the indexed affiliation entities provided by the source databases, related entities such as Peking Union Medical College Hospital, Chinese Academy of Medical Sciences and Peking Union Medical College, and Peking Union Medical College should not be interpreted as fully independent institutional units. The institutional results therefore describe indexed affiliation patterns rather than a fully normalized ranking of independent institutions.

**Table 3:** Top 10 most relevant affiliations in pancreatitis and gut microbiota research.

Rank	Affiliation	Country	Publications
1	PEKING UNION MEDICAL COLLEGE HOSPITAL	China	110
2	DALIAN MEDICAL UNIVERSITY	China	71
3	SHANGHAI JIAO TONG UNIVERSITY	China	67
4	JIANGNAN UNIVERSITY	China	44
5	CHINESE ACADEMY OF MEDICAL SCIENCES - PEKING UNION MEDICAL COLLEGE	China	42
6	PEKING UNION MEDICAL COLLEGE	China	37
7	UNIVERSITÉ PARIS-SACLAY	France	35
8	CAPITAL MEDICAL UNIVERSITY	China	33
9	NANCHANG UNIVERSITY	China	29
10	UNIVERSITY OF MICHIGAN MEDICAL SCHOOL	USA	28

### 3.5 Citation analysis of articles and references

**Figure 6:**
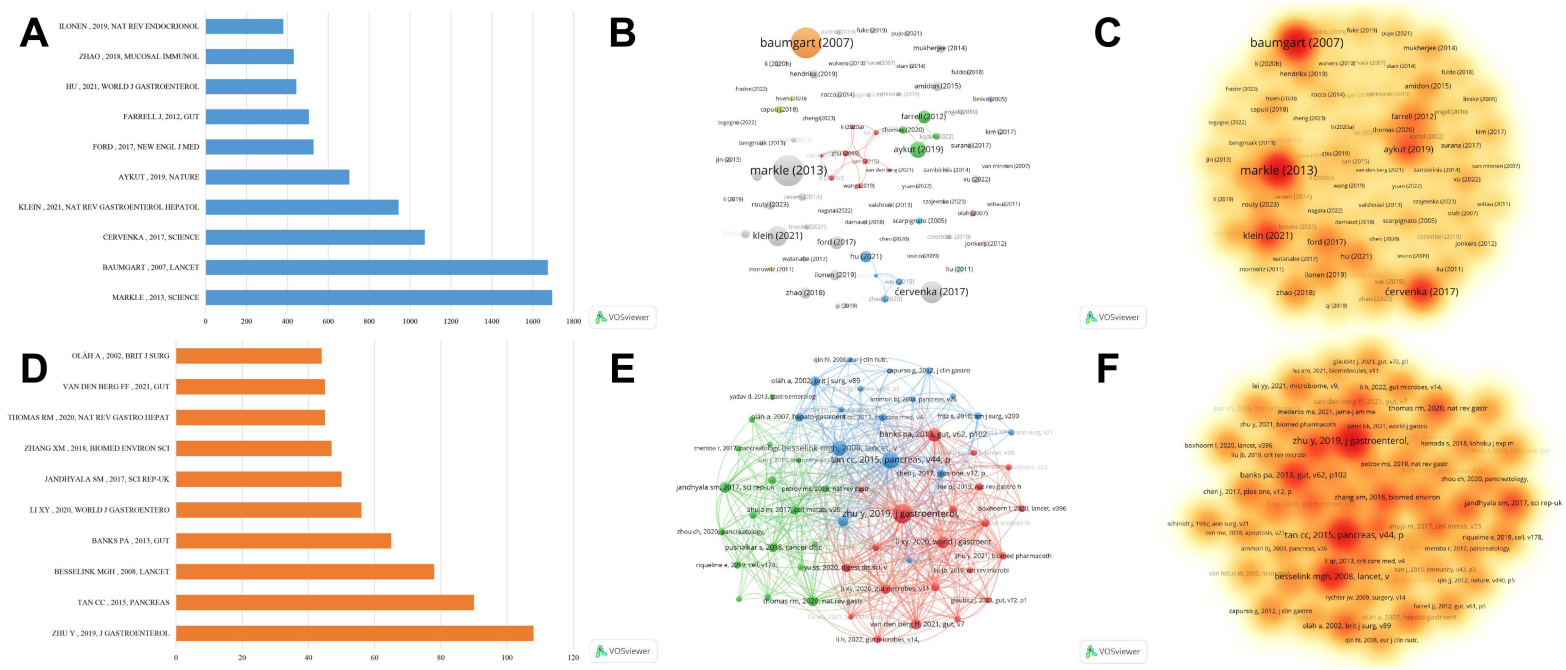
Citation and co-citation analysis of influential articles and references. **(A)** Top 10 co-cited references in the field. **(B)** Co-citation network map of cited references. **(C)** Density map of co-cited references. **(D)** Top 10 highly cited articles. **(E)** Citation network map of highly cited articles. **(F)** Density map of highly cited articles.

The co-cited reference analysis identified several references with high influence in this field (**Figure 6A**; **Table 4**). Markle et al. (2013) (2013, *Science*) ranked first among the top co-cited references, followed by Baumgart et al (Baumgart and Sandborn, 2007). (2007, *Lancet*). Cervenka et al. (2017) (2017, *Science*) and Klein et al (Klein, 2021). (2021, *Nature Reviews Gastroenterology and Hepatology*) also showed high co-citation counts. Other highly co-cited references included Aykut et al. (2019) (2019, *Nature*), Ford et al. (2017) (2017, *New England Journal of Medicine*), Farrell et al. (2012) (2012, *Gut*), Hu et al. (2021) (2021, *World Journal of Gastroenterology*), Zhao et al. (2018) (2018, *Mucosal Immunology*), and Ilonen et al. (2019) (2019, *Nature Reviews Endocrinology*).

**Table 4:** Top 10 co-cited references in pancreatitis and gut microbiota research.

Rank	First author	Article title	Journal	Publication year	Total citations
1	MARKLE	Sex differences in the gut microbiome drive hormone-dependent regulation of autoimmunity	SCIENCE	2013	1695
2	BAUMGART	Inflammatory bowel disease: clinical aspects and established and evolving therapies	LANCET	2007	1674
3	ĆERVENKA	Kynurenines: Tryptophan’s metabolites in exercise, inflammation, and mental health	SCIENCE	2017	1072
4	KLEIN	Pancreatic cancer epidemiology: understanding the role of lifestyle and inherited risk factors	NAT REV GASTROENTEROL HEPATOL	2021	944
5	AYKUT	The fungal mycobiome promotes pancreatic oncogenesis via activation of MBL	NATURE	2019	703
6	FORD	Irritable Bowel Syndrome	NEW ENGL J MED	2017	528
7	FARRELL	Variations of oral microbiota are associated with pancreatic diseases including pancreatic cancer	GUT	2012	505
8	HU	Pancreatic cancer: A review of epidemiology, trend, and risk factors	WORLD J GASTROENTEROL	2021	444
9	ZHAO	GPR43 mediates microbiota metabolite SCFA regulation of antimicrobial peptide expression in intestinal epithelial cells via activation of mTOR and STAT3	MUCOSAL IMMUNOL	2018	431
10	ILONEN	The heterogeneous pathogenesis of type 1 diabetes mellitus	NAT REV ENDOCRIONOL	2019	380

In the co-citation network of cited references, Markle (2013), Baumgart (2007), Cervenka (2017), Aykut (2019), and Klein (2021) occupied relatively prominent positions (**Figure 6B**). The co-citation density map showed concentrated link areas around Markle (2013) and Baumgart (2007), with additional dense areas around Cervenka (2017), Klein (2021), Aykut (2019), Ford (2017), and Zhao (2018) (**Figure 6C**). This pattern indicates that these references were frequently connected within the co-citation network, although not all of them were directly focused on pancreatitis.

Among the highly cited articles, Zhu et al. (2018) (2019, Journal of Gastroenterology) had the highest citation count, followed by Tan et al. (2015) (2015, *Pancreas*) and Besselink et al. (2008) (2008, *Lancet*) (**Figure 6D** and **Table 5**). Banks et al. (2013) (2013, *Gut*), Li et al. (2020) (2020, *World Journal of Gastroenterology*), Jandhyala et al. (2017) (2017, *Scientific Reports*), Zhang et al (Mei et al., 2018). (2018, *Biomedical and Environmental Sciences*), Thomas et al (Thomas and Jobin, 2020). (2020, *Nature Reviews Gastroenterology and Hepatology*), van den Berg et al. (2021) (2021, *Gut*), and Oláh et al. (2007) (2002, *British Journal of Surgery*) were also among the top cited articles.

**Table 5:** Top 10 highly cited articles in pancreatitis and gut microbiota research.

Rank	First author	Article title	Journal	Publication year	Total citations
1	ZHU	Gut microbiota dysbiosis worsens the severity of acute pancreatitis in patients and mice	J GASTROENTEROL	2019	108
2	TAN	Dysbiosis of Intestinal Microbiota Associated With Inflammation Involved in the Progression of Acute Pancreatitis	PANCREAS	2015	90
3	BESSELINK	Probiotic prophylaxis in predicted severe acute pancreatitis: a randomized, double-blind, placebo-controlled trial	LANCET	2008	78
4	BANKS	Classification of acute pancreatitis--2012: revision of the Atlanta classification and definitions by international consensus	GUT	2013	65
5	LI	Role of gut microbiota on intestinal barrier function in acute pancreatitis	WORLD J GASTROENTERO	2020	56
6	JANDHYALA	Altered intestinal microbiota in patients with chronic pancreatitis: implications in diabetes and metabolic abnormalities	SCI REP-UK	2017	50
7	ZHANG	Intestinal Microbial Community Differs between Acute Pancreatitis Patients and Healthy Volunteers	BIOMED ENVIRON SCI	2018	47
8	THOMAS	Microbiota in pancreatic health and disease: the next frontier in microbiome research	NAT REV GASTRO HEPAT	2020	45
9	VAN DEN BERG	Western-type diet influences mortality from necrotizing pancreatitis and demonstrates a central role for butyrate	GUT	2021	45
10	OLÁH	Randomized clinical trial of specific lactobacillus and fiber supplement to early enteral nutrition in patients with acute pancreatitis	BRIT J SURG	2002	44

In the citation network of highly cited articles, Zhu (2019), Tan (2015), and Besselink (2008) were represented by larger nodes, and Banks (2013) and Jandhyala (2017) were also located in relatively central positions (**Figure 6E**). The citation density map showed concentrated areas around Zhu (2019), Tan (2015), and Besselink (2008), while Banks (2013), Li (2020), and Jandhyala (2017) also formed visible hotspot areas (**Figure 6F**). These articles represent highly cited pancreatitis-focused studies related to gut microbiota dysbiosis, acute pancreatitis severity, probiotic intervention, disease classification, intestinal barrier function, and chronic pancreatitis-associated microbial changes.

**Figure 7:**
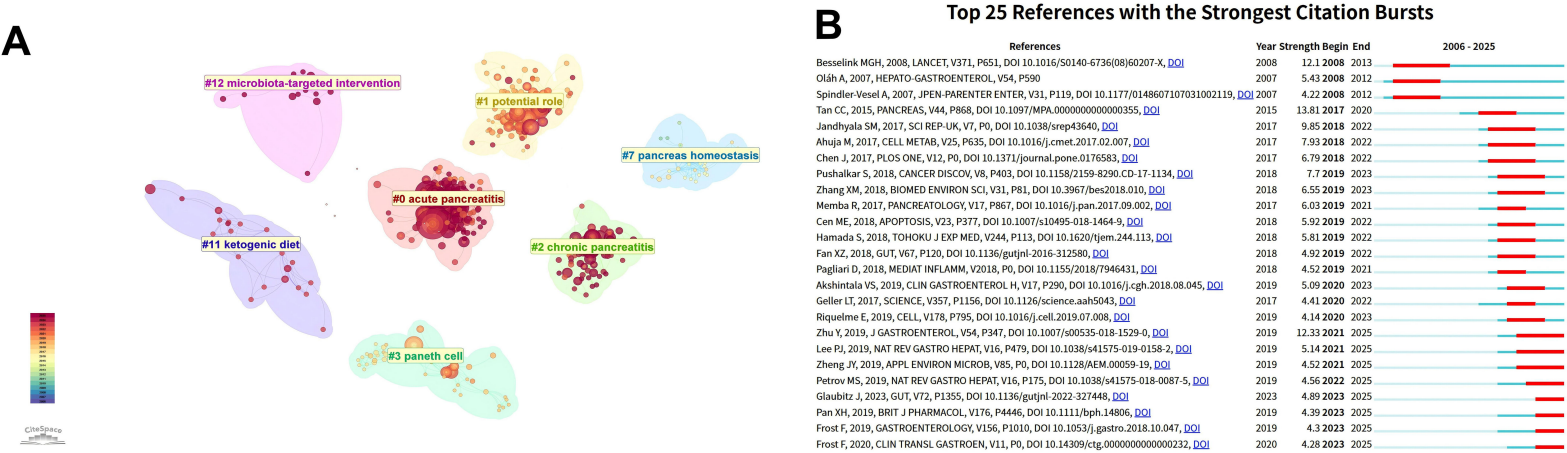
Co-cited reference clustering and citation burst analysis in pancreatitis and gut microbiota research. **(A)** Clusters of key terms in co-cited references. **(B)** Top 25 references with the strongest citation bursts from 2006 to 2025.

The clustering analysis of co-cited references identified several major thematic groups in this field (**Figure 7A**). The largest cluster was #0 acute pancreatitis, which was located near the center of the network and contained a large number of closely connected references. Other major clusters included #1 potential role, #2 chronic pancreatitis, and #3 Paneth cell. Smaller clusters, such as #7 pancreas homeostasis, #11 ketogenic diet, and #12 microbiota-targeted intervention, were also observed. These clusters covered topics related to acute pancreatitis, chronic pancreatitis, pancreatic homeostasis, intestinal epithelial function, dietary intervention, and microbiota-based treatment strategies.

The citation burst analysis showed that several references had strong citation bursts during different periods (**Figure 7B**). Among the earliest burst references, Besselink et al. (2008) (2008) had a burst strength of 12.10, lasting from 2008 to 2013. Oláh et al (Hepato-gastroenterology, 2007). (2007) and Spindler-Vesel et al. (2007) (2007) also showed early citation bursts from 2008 to 2012. In the middle period, Tan et al. (2015) (2015) showed the strongest burst, with a burst strength of 13.81 from 2017 to 2020. Other references with strong bursts during this period included Jandhyala et al. (2017) (2017), Ahuja et al. (2017) (2017), Chen et al (Chen J. et al., 2017). (2017), and Pushalkar et al. (2018) (2018).

Several references showed sustained citation bursts in recent years. Zhu et al. (2021) (2019) had a burst strength of 12.33, with the burst period extending from 2021 to 2025. Lee et al (Lee and Papachristou, 2019). (2019), Zheng et al. (2019) (2019), and Petrov et al (Petrov and Yadav, 2019). (2019) also had citation bursts continuing to 2025. More recent burst references included Glaubitz et al. (2023) (2023), Pan et al. (2019) (2019), and two studies by Frost et al (Frost et al., 2019; Frost et al., 2020). (2019, 2020), all of which showed citation bursts from 2023 to 2025.

### 3.6 Keyword co-occurrence, thematic clustering, and temporal evolution

**Figure 8:**
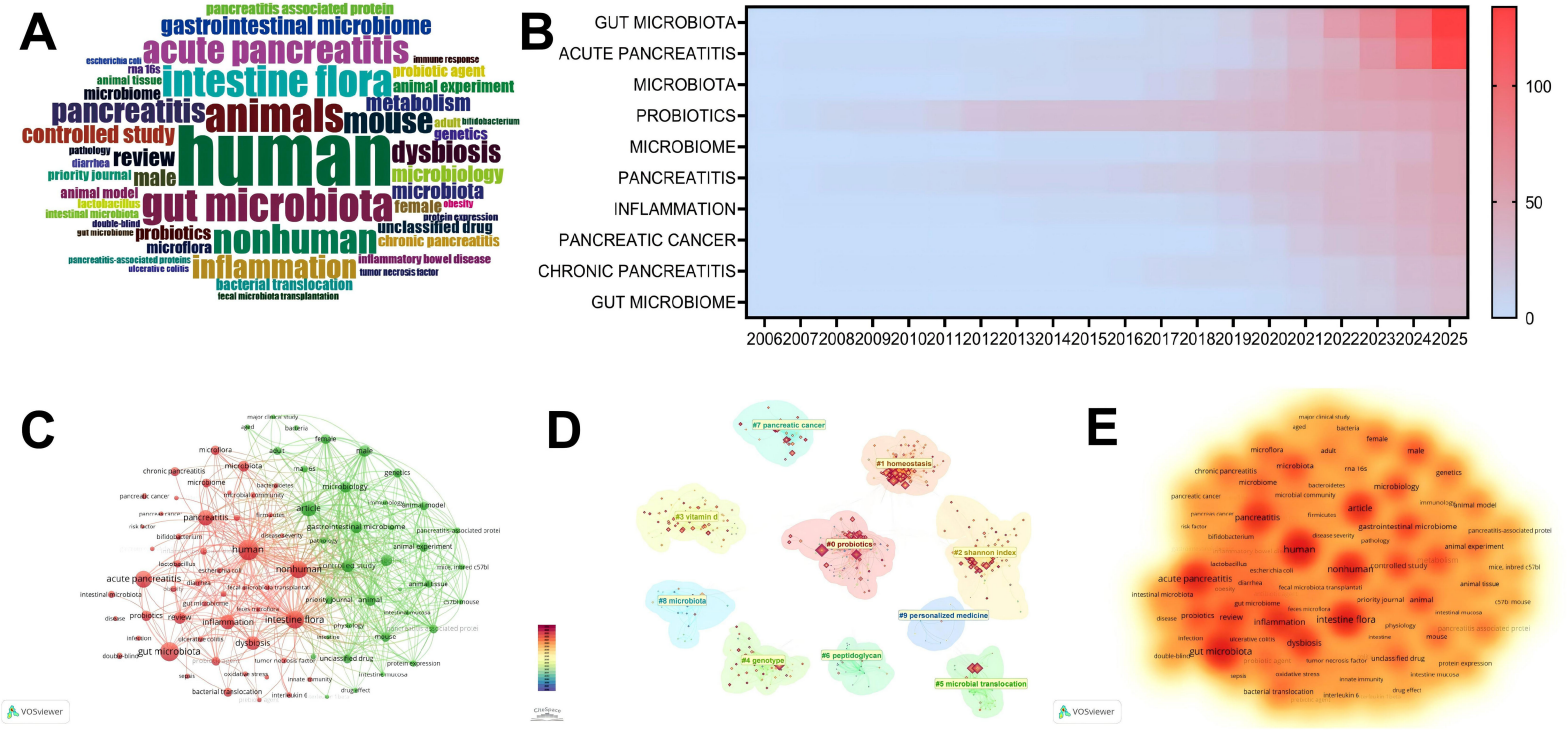
Keyword co-occurrence, clustering, and temporal evolution analysis of pancreatitis and gut microbiota research. **(A)** Keyword word cloud. **(B)** Annual distribution of major keywords. **(C)** Keyword co-occurrence network map. **(D)** Keyword clustering map. **(E)** Keyword density map.

The keyword word cloud summarized the main terms used in pancreatitis and gut microbiota research (**Figure 8A**). The keyword “human” was the most frequently occurring term, followed by gut microbiota, nonhuman, intestine flora, animals, and acute pancreatitis. Other commonly occurring terms included pancreatitis, inflammation, gastrointestinal microbiome, dysbiosis, mouse, probiotics, microbiology, and chronic pancreatitis. Because several high-frequency terms were generic indexing terms, they were interpreted as indicators of database indexing and study type rather than as core research themes.

The annual keyword distribution showed how keyword use changed from 2006 to 2025 (**Figure 8B**). Gut microbiota and acute pancreatitis were the most frequently used terms in recent years. Microbiota, probiotics, microbiome, pancreatitis, and inflammation also showed increasing frequencies over time. In the later years, pancreatic cancer, chronic pancreatitis, and gut microbiome appeared more often than in the early period.

The keyword co-occurrence network suggested that the field was organized around several interconnected themes, including clinical pancreatitis research, gut microbiota dysbiosis, inflammatory response, experimental models, and microbiota-targeted intervention (**Figure 8C**). Terms such as human, gut microbiota, acute pancreatitis, intestine flora, inflammation, and microbiome occupied central positions in this network.

Keyword clustering suggested several major research themes in this field (**Figure 8D**). The main clusters were related to probiotics, homeostasis, alternative medicine, side effects, genotype, microbial metabolites, peptidoglycan, pancreatic cancer, microbiota, and personalized medicine. In the keyword density map, high-density areas were mainly concentrated around human, gut microbiota, nonhuman, acute pancreatitis, intestine flora, inflammation, and dysbiosis (**Figure 8E**).

**Figure 9:**
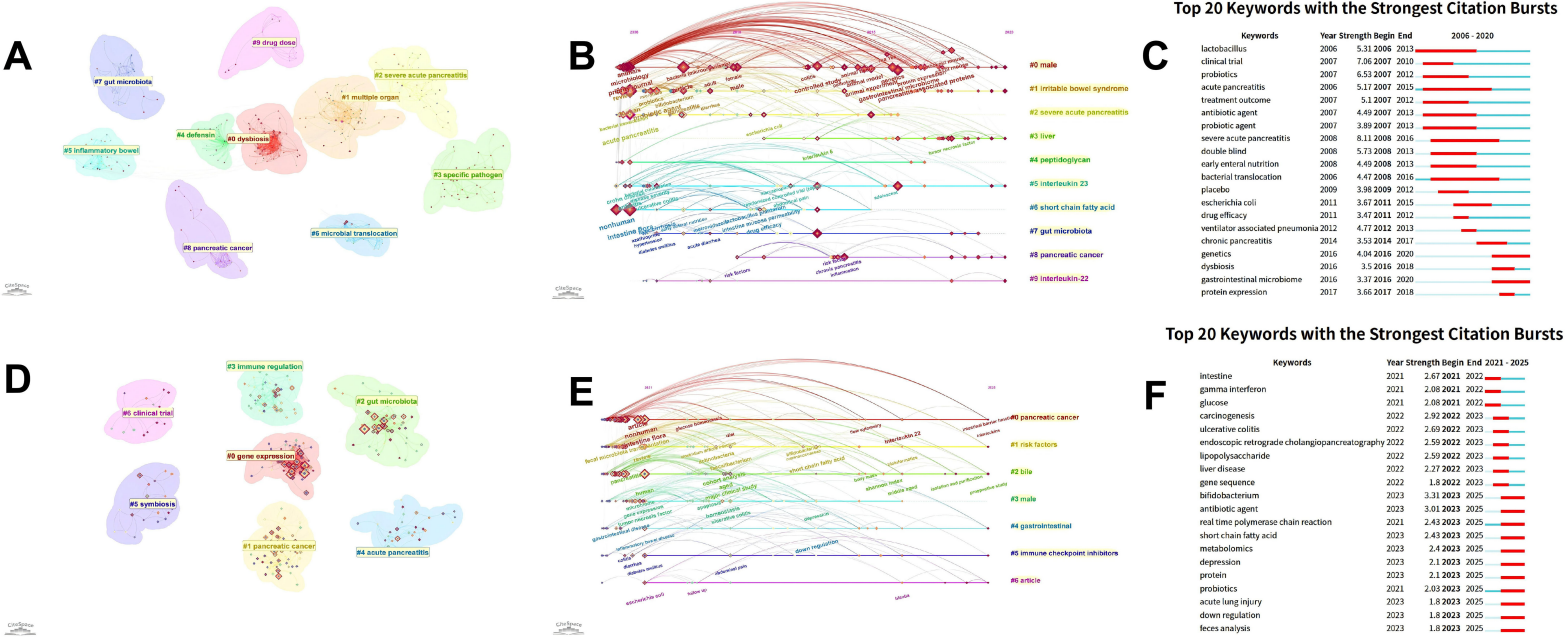
Comparison of thematic structure and temporal evolution of pancreatitis and gut microbiota research before and after 2020. **(A)** Keyword clustering map for 2006-2020. **(B)** Keyword timeline view for 2006-2020. **(C)** Top 20 keywords with the strongest citation bursts for 2006-2020. **(D)** Keyword clustering map for 2021-2025. **(E)** Keyword timeline view for 2021-2025. **(F)** Top 20 keywords with the strongest citation bursts for 2021-2025.

To further characterize temporal changes in the field, we compared the pre-2020 and post-2020 periods using keyword clustering, timeline visualization, and burst detection (**Figures 9A–F**; **Table 6**; **Supplementary Table 2**). In the earlier period (2006-2020), the thematic structure was mainly organized around dysbiosis, severe acute pancreatitis, multiple organ involvement, specific pathogens, microbial translocation, and probiotic-related intervention. The strongest burst keywords in this period included lactobacillus, clinical trial, probiotics, acute pancreatitis, severe acute pancreatitis, antibiotic agent, probiotic agent, early enteral nutrition, bacterial translocation, and chronic pancreatitis (**Figure 9C**).

**Table 6:** Representative burst keywords and their temporal characteristics in pancreatitis and gut microbiota research from 2006 to 2025.

Keyword	Year	Strength	Begin	End	Period
lactobacillus	2006	5.31	2006	2013	2006-2020
clinical trial	2007	7.06	2007	2010	2006-2020
probiotics	2007	6.53	2007	2012	2006-2020
acute pancreatitis	2006	5.17	2007	2015	2006-2020
antibiotic agent	2007	4.49	2007	2013	2006-2020
severe acute pancreatitis	2008	8.11	2008	2016	2006-2020
early enteral nutrition	2008	4.49	2008	2013	2006-2020
bacterial translocation	2006	4.47	2008	2016	2006-2020
chronic pancreatitis	2014	3.53	2014	2017	2006-2020
dysbiosis	2016	3.5	2016	2018	2006-2020
gastrointestinal microbiome	2016	3.37	2016	2020	2006-2020
bifidobacterium	2023	3.31	2023	2025	2021-2025
short-chain fatty acids	2023	2.43	2023	2025	2021-2025
metabolomics	2023	2.4	2023	2025	2021-2025
probiotics	2021	2.03	2023	2025	2021-2025
acute lung injury	2023	1.8	2023	2025	2021-2025
fecal analysis	2023	1.8	2023	2025	2021-2025
lipopolysaccharide	2022	2.59	2022	2023	2021-2025
real-time polymerase chain reaction	2021	2.43	2023	2025	2021-2025
gene sequence	2022	1.8	2022	2023	2021-2025

In contrast, the later period (2021-2025) showed a thematic shift toward gut microbiota, immune regulation, gene expression, symbiosis, acute pancreatitis, and clinical trial-related clusters (**Figures 9D,E**). The strongest burst keywords in this later period included bifidobacterium, short-chain fatty acids, metabolomics, probiotics, acute lung injury, fecal analysis, lipopolysaccharide, and real-time polymerase chain reaction (**Figure 9F**).

Overall, these results suggest a change in research attention from earlier infection-related, probiotic, and clinically oriented topics toward mechanism-oriented and translational themes involving microbial metabolites, host response, and multi-omics investigation.

## 4 Discussion

This bibliometric analysis provides a broad view of research on pancreatitis and gut microbiota from 2006 to 2025. The annual publication trend shows that this field remained small for several years, but entered a period of rapid growth after 2018, with further acceleration after 2020. This change was not only quantitative but also thematic. Earlier studies were more often focused on pancreatic injury, local inflammation, infection, nutrition, and clinical severity, whereas later studies increasingly placed pancreatitis within a wider gut-pancreas framework involving intestinal barrier dysfunction, microbial dysbiosis, immune regulation, metabolic remodeling, and microbiota-derived metabolites. Source analysis supported this shift, as productive and influential journals were distributed not only across gastroenterology and pancreatology, but also across microbiology, immunology, nutrition, and multidisciplinary biomedical science. Together, these findings suggest that pancreatitis and gut microbiota research has developed into a cross-disciplinary field shaped by host-microbe interaction.

The pre-2020 and post-2020 comparison in **Figure 9** and **Table 6** provides additional evidence for this transition. In the earlier period, research was mainly organized around dysbiosis, severe acute pancreatitis, microbial translocation, and probiotic-related intervention, whereas the later period showed a clearer shift toward gut microbiota, immune regulation, gene expression, symbiosis, and acute pancreatitis-related mechanistic themes. The burst keywords also changed accordingly, with early terms emphasizing clinical trials, probiotics, and bacterial translocation, whereas recent terms were more closely linked to bifidobacterium, short-chain fatty acids, metabolomics, and acute lung injury. Together, these bibliometric patterns suggest increasing attention to mechanism-related and translational topics, although they should not be interpreted as direct evidence that the field has entered a distinct developmental stage.

Acute pancreatitis remained the dominant topic in the co-citation clusters, keyword maps, and annual keyword trends. This dominance suggests that, within the retrieved bibliometric dataset, research attention has been concentrated around acute inflammatory injury, disease severity, infection-related complications, and systemic response (Beger and Rau, 2007; Mederos et al., 2021). Importantly, the bibliometric patterns also showed a temporal change within this topic. Earlier work was more closely linked to probiotic or synbiotic intervention, bacterial translocation, and infection control, whereas recent keyword bursts and post-2020 thematic clusters increasingly emphasized dysbiosis, host response, microbial metabolites, and translational mechanisms. Thus, acute pancreatitis remains the central disease context, while the bibliometric patterns suggest increasing attention to mechanism-oriented microbiota research within this context.

Although acute pancreatitis dominated the field, chronic pancreatitis appeared as a distinct and increasingly relevant branch in the co-cited clusters and later keyword trends. This pattern suggests that pancreatitis and gut microbiota research is expanding beyond acute inflammatory injury toward longer-term pancreatic dysfunction and microbe-associated metabolic consequences. Compared with acute pancreatitis, chronic pancreatitis-related studies were less numerous but were more closely connected with exocrine insufficiency, metabolic abnormalities, microbial dysbiosis, and long-term disease burden (Kleeff et al., 2017; Kichler and Jang, 2020; Frost et al., 2021). This distinction helps clarify that the field is not limited to acute severity and infection but is gradually incorporating chronic pancreatic dysfunction into the gut-pancreas research framework.

Some highly co-cited references were not directly focused on pancreatitis, including studies on inflammatory bowel disease, irritable bowel syndrome, type 1 diabetes, pancreatic cancer, and broader microbiome-related biology. This finding should be interpreted in relation to the structure of co-citation analysis. The manual screening criteria were applied to the source records included in the final bibliometric dataset, whereas co-cited references were extracted from the reference lists of these included records. Therefore, non-pancreatitis references could still occupy prominent positions if they were repeatedly cited by pancreatitis and gut microbiota studies as methodological, conceptual, or mechanistic background. These references may reflect shared foundations in microbiome research, host-microbe interaction, immune regulation, inflammation, metabolism, and microbial ecology. At the same time, their prominence may also have been influenced by the broad retrieval strategy, database coverage, and dataset composition. Thus, these references should be interpreted as part of the wider citation environment surrounding pancreatitis and gut microbiota research rather than as the strict core of pancreatitis-specific literature.

The burst keyword analysis provides an additional perspective on the field’s development. Early burst terms were more closely related to clinical intervention, disease severity, infection control, and nutritional support, whereas more recent burst terms were more often associated with microbial taxa, microbial metabolites, molecular detection, and extra-pancreatic complications. This pattern reinforces the view that the field is moving from descriptive and clinically oriented questions toward mechanistic and translational investigation. However, some recent hotspots should still be interpreted cautiously, because several of them are supported mainly by preclinical studies or remain influenced by indexing patterns and terminology variation.

Finally, the geographical and institutional results suggest an uneven global distribution of research activity. China contributed the largest publication output and several leading affiliations, whereas the United States was more prominent in international collaboration. This distribution may reflect both clinical demand and research capacity. At the same time, the collaboration network suggested that many research groups remained relatively clustered, with limited inter-group linkage. For a field that depends heavily on microbiome profiling, heterogeneous study design, sequencing strategy, patient selection, and outcome definition may affect comparability across studies. Greater methodological standardization and broader multicenter collaboration may therefore be important for the next stage of development.

## 5 Limitations

First, the results were dependent on the selected databases and the original retrieval strategy. To improve sensitivity, the search terms were intentionally broad, which may have introduced marginally relevant records at the initial retrieval stage. Although duplicate removal and manual screening were performed to reduce this problem, some degree of selection uncertainty cannot be fully excluded. In addition, because the final dataset relied on manual eligibility screening, borderline records may still have been influenced by subjective judgment.

Second, the combination of WoSCC and Scopus increased database coverage but also introduced challenges related to duplicate identification, differences in indexing practices, and inconsistencies in author names, institutional names, source titles, and database-assigned keywords. These issues may have affected the precision of collaboration, source, and keyword-based analyses.

Third, manual eligibility screening was necessary to exclude studies in which pancreatitis was not a central topic, particularly records related to pancreatic cancer or other gastrointestinal and pancreatic diseases retrieved because of overlapping terminology. Although this step improved topical relevance, some subjective judgment in borderline cases was unavoidable.

Fourth, this study did not include PubMed or DOAJ. Although WoSCC and Scopus provide broad coverage of biomedical and interdisciplinary literature, the exclusion of other databases may still have introduced database selection bias and may have led to omission of some relevant publications, especially records indexed differently across platforms.

Fifth, citation-based indicators favor older publications because they have had more time to accumulate citations. This may partly explain the high average citation values in earlier years. In addition, highly cited references that were not directly focused on pancreatitis may still have influenced the co-citation structure. This pattern may reflect the broader knowledge environment of microbiome, inflammation, metabolism, and host-microbe interaction research, but it may also be partly related to the breadth of the search strategy, database coverage, and dataset composition. Therefore, co-citation clusters should be interpreted as citation-based knowledge structures within the retrieved dataset rather than as a definitive map of pancreatitis-specific mechanistic evidence.

Sixth, keyword and burst analyses are influenced by author keywords, database indexing, generic indexing terms, keyword standardization, and terminology changes over time. Terms such as intestinal flora, microbiota, microbiome, and gut microbiota may refer to overlapping but not fully identical concepts. In addition, generic indexed terms such as “human,” “nonhuman,” “animals,” and “mouse” may reflect indexing categories or study types rather than substantive research themes. More broadly, bibliometric hotspots should be interpreted as indicators of research attention and knowledge structure rather than as direct evidence of biological or clinical importance.

Finally, the findings should be interpreted in light of limitations inherent to bibliometric methods. The observed network structures and thematic patterns may be influenced by database coverage, search strategy design, duplicate removal, manual screening decisions, keyword standardization, and database-specific indexing practices. These factors may affect the apparent hotspots, clusters, and temporal trends identified in this study.

## 6 Conclusion

In conclusion, this bibliometric analysis mapped the global development of research on pancreatitis and gut microbiota from 2006 to 2025. The field showed a rapid increase in publication output after 2018, with China and the United States serving as the main contributors. Acute pancreatitis remained the dominant research theme, while chronic pancreatitis, microbial metabolites, host response, and microbiota-targeted interventions gained increasing attention in recent years. Some peripheral co-citation patterns also reflected broader pancreatic and microbiota-related knowledge domains. Keyword and citation burst analyses suggested increasing research attention to mechanistic and translational topics beyond descriptive dysbiosis. Future studies may require longitudinal clinical cohorts, standardized microbiome sequencing and analysis pipelines, integration of metagenomic and metabolomic data, and carefully designed microbiota-targeted interventions.

## Data Availability

The original contributions presented in the study are included in the article/**Supplementary Material**. Further inquiries can be directed to the corresponding author.
